# Sociodemographic and clinical risk factors for suicidal ideation and suicide attempt in functional/dissociative seizures and epilepsy: a large cohort study

**DOI:** 10.1136/bmjment-2023-300957

**Published:** 2024-04-19

**Authors:** Irene Faiman, John Hodsoll, Iman Jasani, Allan H Young, Paul Shotbolt

**Affiliations:** 1 Department of Psychological Medicine, Institute of Psychiatry, Psychology and Neuroscience, King's College London, London, UK; 2 Bethlem Royal Hospital, South London and Maudsley NHS Foundation Trust, London, UK

**Keywords:** Suicide & self-harm, Substance misuse, Personality disorders, Depression & mood disorders, Anxiety disorders

## Abstract

**Background:**

People with functional/dissociative seizures (FDS) are at elevated suicidality risk.

**Objective:**

To identify risk factors for suicidality in FDS or epilepsy.

**Methods:**

Retrospective cohort study from the UK’s largest tertiary mental healthcare provider, with linked national admission data from the Hospital Episode Statistics. Participants were 2383 people with a primary or secondary diagnosis of FDS or epilepsy attending between 01 January 2007 and 18 June 2021. Outcomes were a first report of suicidal ideation and a first hospital admission for suicide attempt (International Classification of Diseases, version 10: X60–X84). Demographic and clinical risk factors were assessed using multivariable bias-reduced binomial-response generalised linear models.

**Findings:**

In both groups, ethnic minorities had significantly reduced odds of hospitalisation following suicide attempt (OR: 0.45–0.49). Disorder-specific risk factors were gender, age and comorbidity profile. In FDS, both genders had similar suicidality risk; younger age was a risk factor for both outcomes (OR: 0.16–1.91). A diagnosis of depression or personality disorders was associated with higher odds of suicidal ideation (OR: 1.91–3.01). In epilepsy, females had higher odds of suicide attempt-related hospitalisation (OR: 1.64). Age had a quadratic association with both outcomes (OR: 0.88–1.06). A substance abuse disorder was associated with higher suicidal ideation (OR: 2.67). Developmental disorders lowered the risk (OR: 0.16–0.24).

**Conclusions:**

This is the first study systematically reporting risk factors for suicidality in people with FDS. Results for the large epilepsy cohort complement previous studies and will be useful in future meta-analyses.

**Clinical implications:**

Risk factors identified will help identify higher-risk groups in clinical settings.

**WHAT IS ALREADY KNOWN ON THIS TOPIC**

- People with epilepsy and people with functional/dissociative seizures (FDS) are at elevated risk of suicide.
- Identification of risk factors for suicidal ideation and suicide attempt in high-risk groups is essential to inform risk prevention strategies.

**WHAT THIS STUDY ADDS**

- Several factors are associated with suicidality in FDS, including age, ethnicity, comorbid depression and personality disorders.
- In epilepsy, suicidality is associated with age, gender, ethnicity, comorbid substance misuse disorder and developmental disorders.

**HOW THIS STUDY MIGHT AFFECT RESEARCH, PRACTICE OR POLICY**

- While disorder-specific factors will be useful to identify groups at higher risk in clinical settings, general risk factors can be target of population-based preventive strategies.

## Background

Suicide is one of the leading causes of death worldwide, and its prevention has been identified as a ‘global imperative’.^1^ In the UK, the *Five Year Forward View for Mental Health* (2016) declared a national ambition of reducing suicide deaths by 10% by 2020. While national plans have been implemented to advance equality, coverage and access to mental health services, the clinical and academic community should lead in identifying high-risk groups for improved clinical risk assessment and targeted preventive programmes.

Suicidal ideation (thinking about, considering or planning suicide) and suicide attempts (self-harm with intent to die) are strong predictive factors of suicide death; frameworks such as the three-step theory have been proposed to understand the development of ideation and its progression to action.^2^ These immediate precursors of suicide can themselves lead to devastating consequences such as psychological burden, loss of employment, serious injury and disability. As certain factors might have greater or lesser influence in different clinical populations, there is urgent need to understand the risk factors for suicidal ideation and suicide attempts (collectively referred to as ‘suicidality’ hereafter) to recognise people at risk and inform public health strategies.

To address this, the present study focuses on identifying factors associated with suicidality in two groups at elevated suicide risk: people with functional/dissociative seizures (FDS) and people with epilepsy. Epilepsy is a common neurological disorder characterised by an enduring propensity to experience epileptic seizures, that is, transient signs and/or symptoms of excessive or synchronous neuronal activity.^3^ In people with epilepsy, suicide rates are three times those observed in the general population.^4^ FDS are abrupt and observable episodes of altered behaviour or consciousness that superficially resemble epileptic seizures but are not associated with electroencephalography changes.^5^ People with FDS have elevated suicide mortality rates (2.6–18.8%) as compared with the general population,^6–8^ and the presence of FDS has been found to increase the risk of suicide attempt-related hospitalisation in people with epilepsy.^7^

## Objectives

Several risk factors for suicidality in epilepsy have been identified^9–12^; we aim to provide additional evidence from a large cohort for the benefit of future aggregative studies like meta-analyses. On the other hand, evidence on the correlates of suicidality in FDS is scarce, with non-systematic reports from small samples.^13 14^ To the best of our knowledge, this is the first study whose primary aim is to systematically investigate the sociodemographic and clinical factors associated with suicidal ideation and suicide attempt-related hospitalisation in this high-risk population.

## Methods

### Design and setting

This is a retrospective cohort study. Data were extracted by a research nurse using the Clinical Record Interactive Search (CRIS),^15^ a system providing de-identified information from South London and Maudsley National Health Service (NHS) Foundation Trust (SLaM) electronic heath records. SLaM is the largest tertiary mental healthcare provider in Europe, with a catchment of four boroughs in southeast London with a population of over 1.2 million people, hosting the only NHS national specialist tertiary service in the UK offering assessment and treatment of severe dissociative disorders, including FDS.

### Study population

The study’s source population were people attending SLaM between 01 January 2007 and 18 June 2021. Inclusion criteria were a primary or secondary diagnosis of epilepsy (International Classification of Diseases, version 10 (ICD-10) code G40) or FDS (ICD-10: F44.5). We also extracted data for people with concurrent diagnosis of epilepsy and FDS, which were used in a previous study based on the same data^7^; however, the small sample size prevented us from investigating risk and protective factors for this group. Exclusion criteria were a primary or secondary diagnosis of psychotic disorder (ICD-10: F20–F29) or structural brain disease, including cerebral malignancy (ICD-10: C71, C79.3, D43.0–D43.2, D49.6), traumatic brain injury (ICD-10: S01, S02, S06, S07, S09), dementia or progressive neurodegenerative disease (ICD-10: F00–F03).

### Outcome variables: suicidal ideation and suicide attempt

The study outcomes were a first report of suicidal ideation and first suicide attempt-related hospitalisation. Information on suicidal ideation was extracted using a Natural Language Processing (NLP) application (app), using Generalised Architecture for Text Engineering and TextHunter software.^16^ This has 87.8% precision and 91.7% recall to an instance of ideation.^16^ To measure recall of the NLP app specifically for our sample, two researchers (IF, IJ) independently reviewed all 616 text snippets resulting in a positive ideation instance. It was coded whether this was a true and present instance (ie, active within 1 year before the snippet date) or a true past instance (ie, ever before the snippet date). Any disagreements were resolved upon discussion. Recall for present and past instances was 60% and 71%, respectively. Inter-rater agreement was almost perfect (95.3%, Cohen’s kappa=0.90). False positives were corrected to achieve higher accuracy in subsequent analyses.

Information on suicide attempt-related admissions was extracted from Hospital Episode Statistics (HES) admission data linked to our cohort. HES collates national-level data routinely collected by NHS providers (http://www.hscic.gov.uk/hes). People with a first UK-registered hospital admission following suicide attempt (ICD-10: X60–X84) were considered cases of suicide attempt. Due to CRIS data access permissions, HES data were not extracted for patients under 18 years at the time of data collection, and for those who were only ever seen in SLaM Hospital NHS Foundation Trust while under the age of 18 years.

### Sociodemographic and clinical variables

The following hospital-coded information was extracted: age, gender, ethnicity and presence of comorbid neuropsychiatric diagnoses. Age at suicidal ideation and at suicide attempt was calculated; in the absence of suicidality, the age reported was the age at data extraction (18 June 2021) or age at death if deceased. Ethnicity was subdivided into white and other ethnicities (including Asian, black, mixed, multiple and other ethnicities). People were coded as having any neuropsychiatric diagnosis if at least one of the following disorders was recorded before the date of suicidal ideation or suicide attempt (or at any time if suicidality was not observed): substance misuse disorders (ICD-10: F10–F19), anxiety and stress-related disorders (including obsessive compulsive disorder and post-traumatic stress disorder; ICD-10: F40–F42, F43.1), bipolar disorder (ICD-10: F31) or depressive disorders (ICD-10: F32–F33), personality disorder (ICD-10: F60), pervasive developmental disorders or moderate-to-profound intellectual disability (ICD-10: F71–F73, F84).

### Statistical analyses

We explored the association between sociodemographic and clinical factors and study outcomes separately for people with FDS and epilepsy. People were included independently of the diagnosis date. Our sample therefore includes people with a ‘lifetime diagnosis’, under the assumption that both disorders are associated with a neurobiological and neuropsychiatric phenotype existing even before seizure expression and diagnosis. To obtain an estimate of the total effect for each study variable, we first created causal diagrams describing the relationship between variables and outcomes, as recommended^17^ (online supplemental figures 1–4). These were built based on the clinical and academic expertise of a consultant neuropsychiatrist and a consultant psychiatrist (PS and AY). The diagrams were then used to guide variable entry in multivariable bias-reduced binomial-response generalised linear models^18^ using the D3 likelihood ratio test for model comparison with multiply imputed data.^19^ Age was assessed for non-linearity using restricted cubic splines,^20^ where models with zero (linear) to five knots were compared in terms of Akaike Information Criterion (AIC). Lowest AIC were for three knots in epilepsy and four in FDS (locations indicated in the regression tables). Covariates for each model are specified as footnotes to the results tables. As a rule of thumb, analyses were only performed for variables having at least five supporting observations per cell in cross-tabulation with the outcome.^21^ ORs and associated CIs were derived as a measure of association. Tjur’s coefficient of determination (pseudo-R^2^) was used to quantify the amount of shared variance between variables and outcomes.^22^ Significance level was 0.05. Data were analysed using R statistical software^23^ V.4.2, with the ‘brglm’ (V.0.72)^24^ and ‘mice’ (V.3.15) package.^25^

10.1136/bmjment-2023-300957.supp1 Supplementary data

## Findings

### Sociodemographic and clinical characteristics of people with epilepsy

1343 people with epilepsy were included. 12.7% reported suicidal ideation, while 6.1% had a hospital admission following suicide attempt (table 1). There was a similar proportion of males and females, and the predominant ethnicity was white. 57% had at least one neuropsychiatric comorbidity, the most common being pervasive developmental disorders or intellectual disability (31.9%). Other common comorbidities were anxiety or stress-related disorders (12%), and depression or bipolar disorders (12%).

**Table 1:** Sociodemographic and clinical characteristics of people with epilepsy and people with FDS seen at South London and Maudsley Hospital between 01 January 2007 and 18 June 2021

	Epilepsy			FDS		
	All	Suicidal ideation (SI)	Suicide attempt (SA)	All	SI	SA
Total	1343 (100)	171 (12.7)	82 (6.1)	1040 (100)	182 (17.5)	129 (12.4)
Age at SI/SA (if no SI/SA, age at data extraction or death)						
Age, mean (SD)	37.44 (19.14)	33.56 (16.12)	34.09 (14.16)	40.36 (15.17)	33.49 (13.21)	28.24 (11.74)
Gender						
Male	745 (55.5)	86 (11.5)	36 (4.8)	262 (25.2)	39 (14.9)	32 (12.2)
Female	598 (44.5)	85 (14.2)	46 (7.7)	778 (74.8)	143 (18.4)	97 (12.5)
Ethnicity						
White	819 (61.0)	112 (13.7)	65 (7.9)	705 (67.8)	115 (16.3)	102 (14.5)
Asian, black, mixed, other	348 (25.9)	46 (13.2)	12 (3.4)	163 (15.7)	49 (30.1)	11 (6.7)
Missing	176 (13.1)	13 (7.4)	5 (2.8)	172 (16.5)	18 (10.5)	16 (9.3)
Any neuropsychiatric comorbidities						
No	581 (43.3)	98 (16.9)	60 (10.3)	836 (80.4)	133 (15.9)	116 (13.9)
Yes	762 (56.7)	73 (9.6)	22 (2.9)	204 (19.6)	49 (24.0)	13 (6.4)
Depressive disorders/bipolar disorder						
No	1183 (88.1)	150 (12.7)	72 (6.1)	921 (88.6)	151 (16.4)	109 (11.8)
Yes	160 (11.9)	21 (13.1)	10 (6.2)	119 (11.4)	31 (26.0)	20 (16.8)
Anxiety and stress-related disorders						
No	1178 (87.7)	152 (12.9)	75 (6.4)	952 (91.5)	163 (17.1)	125 (13.1)
Yes	165 (12.3)	19 (11.5)	7 (4.2)	88 (8.5)	19 (21.6)	4 (4.5)
Substance misuse disorders						
No	1269 (94.5)	151 (11.9)	77 (6.1)	1022 (98.3)	180 (17.6)	127 (12.4)
Yes	74 (5.5)	20 (27.0)	5 (6.7)	18 (1.7)	2 (11.1)	2 (11.1)
Personality disorder						
No	1329 (99.0)	170 (12.8)	81 (6.1)	1012 (97.3)	171 (16.9)	125 (12.3)
Yes	14 (1.0)	1 (7.1)	1 (7.1)	28 (2.7)	11 (39.3)	4 (14.3)
Pervasive developmental disorders/intellectual disability						
No	915 (68.1)	152 (16.6)	76 (8.3)	1029 (98.9)	181 (17.6)	127 (12.3)
Yes	428 (31.9)	19 (4.4)	6 (1.4)	11 (1.1)	1 (9.1)	2 (18.2)
Previous SA (if no SI, at any time)						
No	1269 (94.5)	142 (11.2)	–	923 (88.7)	156 (16.9)	–
Yes	74 (5.5)	29 (39.2)	–	117 (11.3)	26 (22.2)	–
Previous SI (if no SA, at any time)						
No	1176 (87.6)	–	74 (6.3)	850 (81.7)	–	117 (13.8)
Yes	167 (12.4)	–	8 (4.8)	190 (18.3)	–	12 (6.3)

Reported are number of cases (percentages) unless otherwise specified.

FDS, functional/dissociative seizures.

### Sociodemographic and clinical characteristics of people with FDS

1040 people with FDS were included. 17.5% reported suicidal ideation, while 12.4% had a hospital admission following suicide attempt (table 1). 75% were females, and the predominant ethnicity was white. A neuropsychiatric comorbidity was present in approximately 20%, the most common being depression or bipolar disorder (11%) and anxiety and stress-related disorders (8.5%).

### Risk and protective factors in people with epilepsy

**Figure 1:**
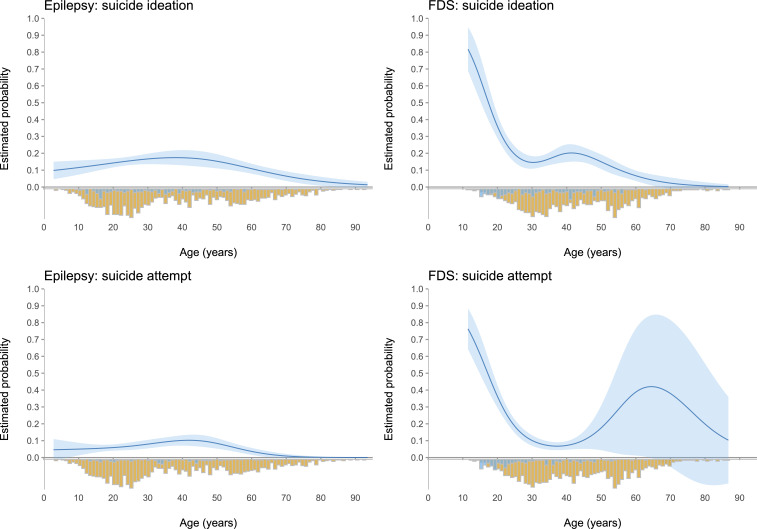
Loess smoothed (local non-linear regression) curves showing probability of suicidal ideation and suicide attempt-related hospitalisation by age per each diagnostic group. (Top left) Probability of suicidal ideation by age in epilepsy; (bottom left) probability of suicide attempt-related admission by age in epilepsy; (top right) probability of suicidal ideation by age in FDS; (bottom right) probability of suicide attempt-related admission by age in FDS. On the negative side of the y axis are depicted the age distributions (number of cases) stratified by presence of outcome (blue: suicidal ideation/suicide attempt present; yellow: suicidal ideation/suicide attempt absent). FDS, functional/dissociative seizures.

Results of the bias-reduced logistic regression indicated that in people with epilepsy, there is a significant non-linear association between age and suicidal ideation; odds were stable until the knot location (age 33 years; OR: 1.03; 95% CI: 1.00, 1.05) before declining (OR: 0.94; 95% CI: 0.90, 0.98; table 2 and figure 1). Age had a significant quadratic association with suicide attempt-related admission, with risk increasing until age 33/knot before declining (table 3 and figure 1). Both genders had similar odds of experiencing suicidal ideation (OR: 1.27; 95% CI: 0.92, 1.75). For suicide attempt, the odds of females were significantly higher than males (OR: 1.64; 95% CI: 1.05, 2.56). Ethnicity was not associated with suicidal ideation (OR: 0.96; 95% CI: 0.66, 1.38). The odds of people of white ethnicity being admitted following suicide attempt were significantly higher (1/0.45=2.22 times) as compared with other ethnic backgrounds (Asian, black, mixed, multiple and other; OR: 0.45; 95% CI: 0.24, 0.85). The odds of people with a substance misuse disorder of experiencing suicidal ideation were 2.67 times the odds of people without (OR: 2.67; 95% CI: 1.61, 4.74). The presence of a pervasive developmental disorder or intellectual disability was associated with a significantly reduced likelihood of suicidal ideation (OR: 0.24; 95% CI: 0.15, 0.39) and suicide attempt-related admission (OR: 0.16; 95% CI: 0.07, 0.35). There was no association between outcomes and a diagnosis of depression or bipolar disorder, or anxiety or stress-related disorders (tables 2 and 3). Reporting suicidal ideation did not affect the likelihood of subsequent suicide attempt-related admission (OR: 1.04; 95% CI: 0.49, 2.18). See online supplemental material for D3 likelihood ratio test. R^2^ values were low; predictor variables explained between 0% and 3.8% of the variance in the outcomes studied (online supplemental tables 1 and 2).

**Table 2:** Results from bias-reduced logistic regression analysis on suicidal ideation (SI) in people with epilepsy, presented as beta values and associated SEs, ORs with 95% CIs and significance value (p)

	B (SE)	OR	95% CI for OR (lower)	95% CI for OR (upper)	P value
Age at SI (linear)*	−0.01 (0.0)	0.99	0.98	1.00	0.008
Age at SI (non-linear 1, age <32.8)*	0.02 (0.01)	1.03	1.00	1.05	0.068
Age at SI (non-linear 2, age >32.8)*	−0.06 (0.02)	0.94	0.9	0.98	0.004
Gender†	0.24 (0.16)	1.27	0.92	1.75	0.145
Ethnicity‡	−0.04 (0.19)	0.96	0.66	1.38	0.815
Any neuropsychiatric comorbidities§	−0.64 (0.17)	0.53	0.38	0.73	<0.001
Depression/bipolar¶	0.06 (0.25)	1.06	0.64	1.73	0.83
Anxiety/stress related**	−0.12 (0.26)	0.89	0.53	1.47	0.64
Substance misuse††	1.02 (0.28)	2.67	1.61	4.74	<0.001
PDD/ID‡‡	−1.43 (0.25)	0.24	0.15	0.39	<0.001

*Model just including age as a predictor.

†Model just includes gender as a predictor, with male as reference category.

‡Model just includes ethnicity as a predictor, with white as reference category.

§Model includes ethnicity and gender as covariates.

¶Model including ethnicity, gender, anxiety and depressive disorder/ID as covariates.

**Model including ethnicity, gender, depressive disorders and substance misuse as covariates.

††Model including ethnicity and anxiety disorders as covariates.

‡‡Model only including PDD/ID as predictor.

ID, intellectual disability; PDD, pervasive developmental disorder.

**Table 3:** Results from bias-reduced logistic regression analysis on suicide attempt (SA)-related admissions in people with epilepsy, presented as beta values and associated SEs, ORs with 95% CIs and significance value (p)

	B (SE)	OR	95% CI for OR (lower)	95% CI for OR (upper)	P value
Age at SA (linear)*	−0.01 (0.01)	0.99	0.98	1.00	0.11
Age at SA (non-linear 1, age <33.2)*	0.06 (0.02)	1.06	1.02	1.11	0.003
Age at SA (non-linear 2, age <33.2)*	−0.13 (0.04)	0.88	0.82	0.94	<0.001
Gender†	0.49 (0.23)	1.64	1.05	2.56	0.03
Ethnicity‡	−0.80 (0.32)	0.45	0.24	0.85	0.01
Any neuropsychiatric comorbidities§	−1.43 (0.25)	0.24	0.15	0.39	<0.001
Depression/bipolar¶	−0.20 (0.34)	0.82	0.42	1.60	0.56
Anxiety/stress related**	−0.56 (0.39)	0.57	0.27	1.23	0.15
Substance misuse††	0.09 (0.46)	1.09	0.44	2.68	0.85
PDD/ID‡‡	−1.85 (0.41)	0.16	0.07	0.35	<0.001
Previous SI§§	0.03 (0.38)	1.04	0.49	2.18	0.93

*Model just including age as a predictor.

†Model just includes gender as a predictor, with male as reference category.

‡Model just includes ethnicity as a predictor, with white as reference category.

§Model includes ethnicity and gender as covariates.

¶Model including ethnicity, gender, anxiety disorders and PDD/ID as covariates.

**Model including ethnicity, gender, depressive disorders and substance misuse as covariates.

††Model including ethnicity and anxiety disorders as covariates.

‡‡Model only including PDD/ID as predictor.

§§Model includes any psychiatric comorbidities, ethnicity and gender as covariates.

ID, intellectual disability; PDD, pervasive developmental disorder; SI, suicidal ideation.

### Risk and protective factors in people with FDS

Results of the bias-reduced logistic regression indicated that as age of people with FDS increases, there is a significant decrease in the odds of suicidal ideation (up to age 33/knot location: OR: 0.85; 95% CI: 0.81, 0.90) and suicide attempt-related hospitalisation (up to age 32/knot location: OR: 0.81; 95% CI: 0.77, 0.86; figure 1). After age 33 or 32 years, risk increases again until age 47 years before declining (tables 4 and 5 and figure 1). Both genders had similar odds for suicidality (tables 4 and 5). The odds of people of Asian, black, mixed, multiple and other ethnicities of experiencing suicidal ideation were significantly higher (2.4 times) than people of white ethnicity (OR: 2.40; 95% CI: 1.63, 3.53). However, people of white ethnicity were 1/0.49=2.0 times more likely to have an admission following suicide attempt (OR: 0.49; 95% CI: 0.26, 0.92). For suicidal ideation, the odds of people with comorbid depression or bipolar disorder were significantly higher compared with those without these diagnoses (OR: 1.91; 95% CI: 1.22, 3.00). The same was true for comorbid personality disorder (OR: 3.01; 95% CI: 1.37, 6.63). There was no association between suicidal ideation and a diagnosis of anxiety or stress-related disorders (table 4). For suicide attempt, it was only possible to explore the association with a diagnosis of depression or bipolar disorder (non-significant; table 5) due to low cell counts for the other comorbidities. Reporting previous ideation was not associated with subsequent suicide attempt-related admission (OR: 0.62; 95% CI: 0.33, 1.16). Predictors explained between 0% and 15.7% of the variance in the outcomes studied (online supplemental tables 3 and 4).

**Table 4:** Results from bias-reduced logistic regression analysis on suicidal ideation (SI) in people with FDS, presented as beta values and associated SEs, ORs with 95% CIs and significance value (p)

	B (SE)	OR	95% CI for OR (lower)	95% CI for OR (upper)	P value
Age at SI (linear)*	−0.05 (0.01)	0.96	0.94	0.97	<0.001
Age at SI (non-linear 1; age <33)*	−0.16 (0.03)	0.85	0.81	0.9	<0.001
Age at SI (non-linear 2; 33<age<47)*	0.55 (0.11)	1.73	1.41	2.14	<0.001
Age at SI (non-linear 3; age >47)*	−1.59 (0.32)	0.20	0.11	0.38	<0.001
Gender†	0.25 (0.20)	1.28	0.87	1.88	0.21
Ethnicity‡	0.87 (0.20)	2.40	1.63	3.53	<0.001
Any neuropsychiatric comorbidities§	0.52 (0.19)	1.68	1.15	2.45	0.007
Depression/bipolar¶	0.65 (0.23)	1.91	1.22	3.00	0.005
Anxiety/stress related**	0.29 (0.28)	1.34	0.78	2.29	0.29
Personality disorder††	1.10 (0.40)	3.01	1.37	6.63	0.006

*Model just including age as a predictor.

†Model just includes gender as a predictor, with male as reference category.

‡Model just includes ethnicity as a predictor, with white as reference category.

§Model includes ethnicity and gender as covariates.

¶Model including ethnicity, gender, anxiety disorders and PDD/ID as covariates.

**Model including ethnicity, gender, depressive disorders and substance misuse as covariates.

††Model including ethnicity and gender as covariates.

FDS, functional/dissociative seizures; ID, intellectual disability; PDD, pervasive developmental disorder.

**Table 5:** Results from bias-reduced logistic regression analysis on suicide attempt (SA)-related admissions in people with FDS, presented as beta values and associated SEs, ORs with 95% CIs and significance value (p)

	B (SE)	OR	95% CI for OR (lower)	95% CI for OR (upper)	P value
Age at SA (linear)*	−0.08 (0.01)	0.92	0.90	0.94	<0.001
Age at SA (non-linear 1; age <32)*	−0.21 (0.03)	0.81	0.77	0.86	<0.001
Age at SA (non-linear 2; 32<age<47)*	0.65 (0.14)	1.91	1.45	2.52	<0.001
Age at SA (non-linear 3; age >47)*	−1.81 (0.43)	0.16	0.07	0.38	<0.001
Gender†	0.01 (0.22)	1.01	0.66	1.55	0.95
Ethnicity‡	−0.72 (0.32)	0.49	0.26	0.92	0.026
Neuropsychiatric comorbidities					
Depression/bipolar§	0.07 (0.26)	1.07	0.64	1.78	0.79
Previous SI¶	−0.48 (0.32)	0.62	0.33	1.16	0.13

*Model just including age as a predictor.

†Model just includes gender as a predictor, with male as reference category.

‡Model just includes ethnicity as a predictor, with white as reference category.

§Model including ethnicity, gender, anxiety disorders and PDD/ID as covariates.

¶Model includes any psychiatric comorbidities, ethnicity and gender as covariates.

FDS, functional/dissociative seizures; ID, intellectual disability; PDD, pervasive developmental disorder; SI, suicidal ideation.

## Discussion and clinical implications

This work sought to identify the sociodemographic and comorbidity correlates of suicidal ideation and suicide attempt-related hospital admissions in large cohorts of people with FDS or epilepsy. We studied group-specific associations with age, gender, ethnicity and comorbid neuropsychiatric diagnoses. We present novel findings for risk factors in the population with FDS which have not been previously studied.

We identify factors that have similar relationships with suicidality across the two groups, indicating that these are more likely to be general risk factors, rather than specific to a particular clinical population.

For both cohorts, ethnicity accounted for a small proportion of the observed variance in suicide attempt-related hospitalisation (0.5–0.6%). However, a consistent association was observed; people of Asian, black, mixed, multiple or other backgrounds had significantly reduced odds for such hospitalisations (51–55% reduction) compared with people of white ethnicity. These results align with the largest UK community study,^26^ highlighting that people of ethnic minorities are half as likely to receive medical attention following suicide attempt than people of white ethnicity. In our study, this was observed despite ethnic minorities having significantly higher odds of reporting suicidal ideation in part of our sample (FDS group, OR: 2.4). It has been suggested that scarcer service accessibility or fear of stigma may deter individuals from ethnic minorities seeking medical attention after suicide attempts.^26^ Further research is required to understand this; if appropriate, efforts should be implemented to reduce stigmatisation and improve service access for ethnic minorities following suicide attempts.

In both cohorts, reporting suicidal ideation was not associated with a higher likelihood of subsequent suicide attempt-related admission. Our measure of suicidal ideation relied on NLP of clinical notes rather than systematic assessment, and it is uncertain if ideation was reported elsewhere. Nevertheless, our results highlight the difficulty of predicting suicide attempts among people experiencing suicidal ideation, as most do not progress to actual attempts.^27 28^

We have identified a number of risk and protective factors for suicidality that are unique to each diagnostic group.

In people with FDS, both genders had comparable odds of reporting suicidal ideation and of being hospitalised following a suicide attempt. This indicates that although the FDS group has a high prevalence (75%) of females, both genders are at similar suicidality risk. This is a novel finding which has not been previously reported. In people with epilepsy, no gender effect was observed for suicidal ideation; this is in line with results from a large meta-regression^29^ but not another,^4^ indicating the need for additional investigation. However, females with epilepsy had significantly higher odds of having a suicide attempt-related admission (OR: 1.64; 0.4% variance explained), in line with some previous evidence^11 28^ but not another.^29^

In people with FDS, age was significantly associated with a first instance of suicidal ideation and first suicide attempt-related hospitalisation, each explaining 8.8% and 15.7% of the outcome variance, respectively. Consistent with previous studies in the general population,^30^ we observed that adolescence and early adulthood are periods of greatest risk of onset of suicidality in FDS, with risk varying non-linearly as a function of age.

In epilepsy, age had a significant non-linear association with both suicidality outcomes, explaining 1.1–1.2% of their variance. Consistent with the probability distribution observed in this study, previous evidence indicates that people with epilepsy tend to complete suicide later in life (in their 40s) compared with those without epilepsy.^31 32^ This might relate to the highest prevalence of epilepsy occurring in the 35–64 years age group,^32^ although our sample distribution does not support this, as it includes a high proportion of cases under 30 years old. Suicidality in epilepsy is multifaceted and may have distinct underlying mechanisms compared with those driving the suicidality peak observed in adolescence and early adulthood in FDS or the general population. Epilepsy-related and psychosocial factors, treatment responsiveness and the shared neurobiological substrate between depression and epilepsy^33^ may contribute to a more chronic vulnerability to suicidality later in life.

Analysis of five comorbidity classes revealed that suicidality in FDS and epilepsy is associated with a distinct pattern of neuropsychiatric comorbidities.

In people with epilepsy, those with a comorbid diagnosis of substance misuse had 167% higher odds of reporting suicidal ideation (1.1% variance explained), consistently with reports from a recent meta-analysis.^34^ As previously reported,^12^ we found that a strong protective factor against suicidal ideation (76% reduction in odds) and suicide attempt-related admissions (84% reduction) was a diagnosis of moderate-to-profound intellectual disability or pervasive developmental disorder such as autism, Rett syndrome and Asperger syndrome. This explained 2–2.9% of the variance in suicidality outcomes and is the driver of the seemingly ‘protective’ effect of having ‘any neuropsychiatric comorbidity’ observed in results tables. An impaired communication of thoughts and emotions might contribute to under-reported suicidal ideation in populations with pervasive developmental disorder/intellectual disability; these may also have only a partial understanding of the concepts of life and death, which serves as the basis of forming suicide intent.^35^ The reasons driving this finding should be a matter of further study.

In people with epilepsy, diagnoses of depression, bipolar disorder, anxiety or stress-related disorders (including obsessive compulsive disorder and post-traumatic stress disorder) were not associated with suicidality. While meta-analysis results suggest an overall weak association between anxiety, related disorders and suicidality,^36^ our finding of a lack of association for depressive disorders is surprising, as these have been reported as the major risk factor for suicidal ideation in epilepsy.^10^ However, recent meta-analysis results also suggest that depression might not effectively predict suicide attempts.^27^ Our results may be due to sample-specific characteristics, to sampling from a large tertiary mental health hospital or to limitations in capturing relevant factors such as disorder severity or hopelessness using ICD-10 codes for depression.^2 37^ These results should be better understood in future meta-analyses.

In people with FDS, the odds of experiencing suicidal ideation were 200% higher in those with a comorbid diagnosis of personality disorder, and this accounted for 2.9% of the variance. Suicidality is a central feature of the personality disorder subtypes strongly associated with FDS,^38^ such as borderline personality disorder. The risk of reporting suicidal ideation was also higher in the presence of a diagnosis of depression or bipolar disorder (91% increase in odds, 3% variance explained), although this did not translate to an increased risk of suicide attempt-related hospitalisation in this group. Again, as depressive disorders are strongly associated with suicidality, these results do not imply an absence of increased risk and should be considered in future aggregative studies for a more comprehensive interpretation. It was not possible to estimate the association between suicide attempt-related admissions and a comorbid diagnosis of anxiety or stress-related disorders, personality disorders, substance misuse disorder or pervasive developmental disorder due to low numbers in this group.

**Figure 2:**
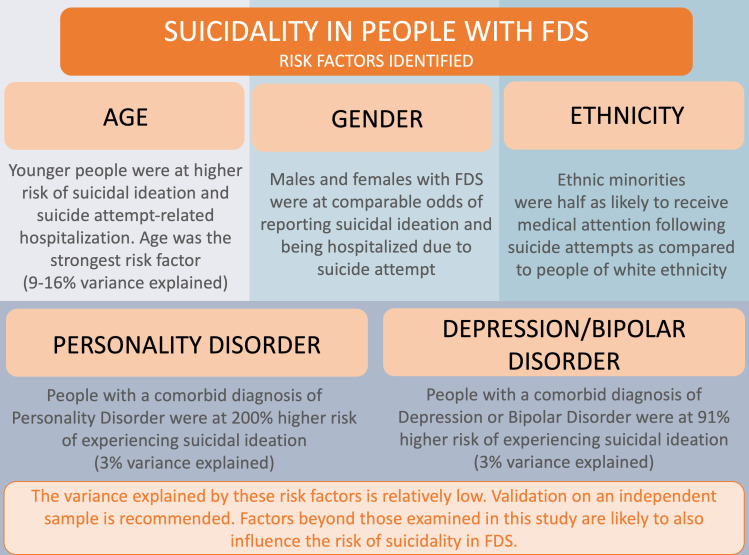
Summary of main findings on risk factors for suicidality in people with FDS. FDS, functional/dissociative seizures.

Figure 2 includes a summary of clinically relevant study findings for the FDS group.

This study’s major strength is the sizeable patient sample (n=2383) from the UK largest tertiary mental health hospital, spanning 15 years. Linkage to national admission data from the HES ensured that hospitalisation could be captured independently from location of the admitting hospital. However, non-admissions following suicide attempt, estimated to be 50% of those attempting suicide,^39^ were not considered. Cases of suicidal ideation were not captured if unreported or undocumented during hospital appointments at our site. Our NLP app for identifying ideation had 60–71% recall and an estimated 91.7% precision; while all false positives were corrected prior to analyses, a proportion of relevant instances might have been missed. Severity and frequency of ideation were not assessed.

Due to the study’s retrospective nature, information on how the diagnoses of epilepsy or FDS were arrived at was not available, and a degree of misdiagnosis might be present; for epilepsy, this is estimated to be between 4.6% and 5.6% following specialist review.^40^ We expect minimal misdiagnosis rates for FDS due to the hospital’s high specialisation for FDS. Our use of ICD-10 codes for identifying comorbid disorders might underestimate their true prevalence.

Our patient cohort might differ from that of community studies because of sampling from a tertiary mental health hospital. For example, our epilepsy sample showed a higher prevalence of at least one of the psychiatric comorbidities under study (57%), compared with the typically reported 30% prevalence for psychiatric comorbidities in epilepsy.^41^ Results may also vary under different theoretical assumptions (diagrams).

## Conclusion

This is the first study to systematically examine the correlates of suicidality in people with FDS. We also examined a large sample of people with epilepsy. Collectively, results suggest that shared sociodemographic and clinical risk factors, as well as disorder-specific risk factors for suicidality, can be identified. Characteristics such as ethnicity had similar, systematic associations with suicidality in both groups. A pattern of association that was unique to each clinical population was found for gender, age and comorbidity profile.

While correlates of suicidality such as disorder-specific comorbidity profiles will be useful to identify groups at higher risk in clinical settings, factors such as ethnicity are suitable targets for population-based health strategies and preventive programmes.

## Data Availability

Data may be obtained from a third party and are not publicly available. Data may be obtained from a third party and are not publicly available. Access to Clinical Record Interactive Search (CRIS) data used for this study is regulated by the CRIS Oversight Committee and the ’Oxfordshire C’ Research Ethics approval for secondary analysis of CRIS data (23/SC/0257). Data used for this research cannot be shared without prior approval from the CRIS Oversight Committee. Those interested should contact Robert Stewart (robert. stewart@kcl.ac.uk), CRIS academic lead.
