# Supplementary material for: Sociodemographic and clinical risk factors for suicidal ideation and suicide attempt in functional/dissociative seizures and epilepsy: a large cohort study
Source: BMJ Ment Health. 2024 Apr 19;27(1):e300957. doi: 10.1136/bmjment-2023-300957 (PMC11033658; doi:10.1136/bmjment-2023-300957)
Supplement: Supplementary data [file bmjment-2023-300957supp001.pdf]

SUPPLEMENTARY FIGURES AND TABLES

Supplementary Figures

Diagrams depicted in Supplementary Figures 1 and 2 were used when estimating the association between our study outcomes and demographic and clinical variables of interest. Diagrams depicted in Supplementary Figures 3 and 4 were used when estimating the association between our study outcomes and each neuropsychiatric comorbidity specifically; this allowed us to further determine the specific drivers of the association between having any neuropsychiatric comorbidity and the study outcomes.

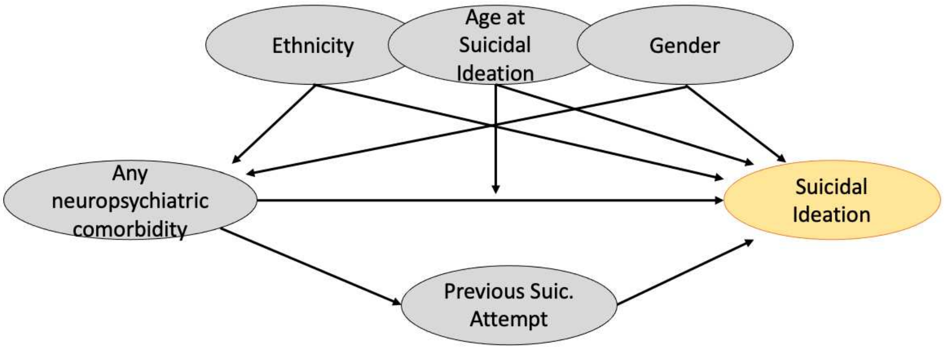

Supplementary figure 1. Causal diagram depicting demographic and clinical variables in relation to Suicidal Ideation. An arrow's direction represents the theoretical direction of causal effect. An arrow pointing to the middle body of another arrow indicates a theoretical moderation effect. Dark grey = predictor variables. Yellow = predicted variable.

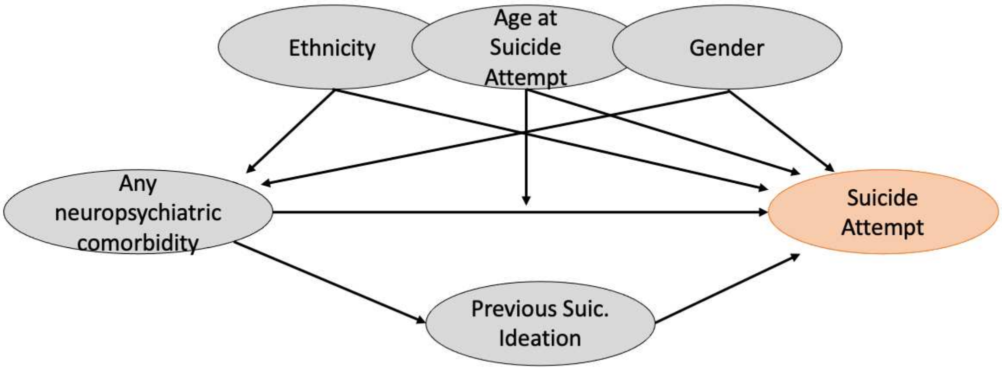

Supplementary figure 2. Causal diagram depicting demographic and clinical variables in relation to Suicide Attempt. An arrow's direction represents the theoretical direction of causal effect. An arrow pointing to the middle body of another arrow indicates a theoretical moderation effect. Dark grey = predictor variables. Red = predicted variable.

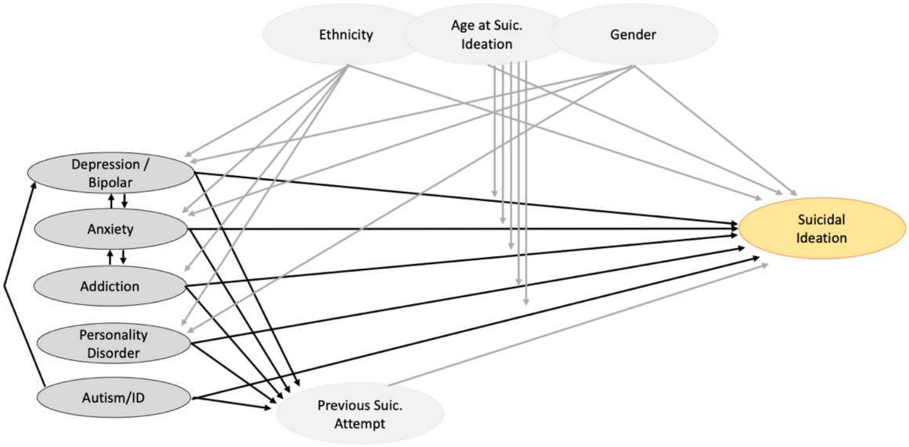

Supplementary figure 3. Causal diagram depicting psychiatric comorbidity variables in relation to Suicidal Ideation. An arrow's direction represents the theoretical direction of causal effect. An arrow pointing to the middle body of another arrow indicates a theoretical moderation effect. Dark grey = predictor variables. Light grey = control variables. Yellow = predicted variable.

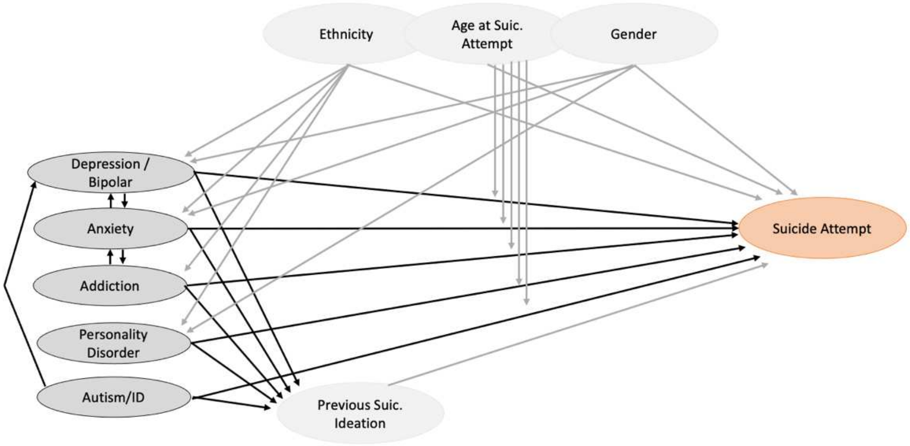

Supplementary figure 4. Causal diagram depicting psychiatric comorbidity variables in relation to Suicide Attempt. An arrow's direction represents the theoretical direction of causal effect. An arrow pointing to the middle body of another arrow indicates a theoretical moderation effect. Dark grey = predictor variables. Light grey = control variables. Red = predicted variable.

Supplementary Tables

Supplementary Table 1 (supplement to main document Table 2). Supplementary results from bias-reduced Logistic Regression analysis on **suicidal ideation in people with epilepsy.** Model fit statistics by target risk factor including the D3 likelihood ratio test for multiply imputed data and average R2 value across imputed data sets.

**Term**	**D3**	**p**	**R2**
Age at SI	7.5 (1, 1728711)	0.006	0.005
Age at SI (non-linear)	8.3 (2, 2131450)	0.000	0.011
Gender: Female (vs Male)	2.1 (1, 1873551)	0.145	0.002
Ethnicity: White, Asian, Black, Mixed, Other (vs White)	0.1 (1, 2260)	0.796	0.000

Term	D3	p	R2
Any neuropsychiatric comorbidities: Yes (vs No)	5.7 (3, 84900)	0.001	0.013
- Depression / Bipolar: Yes (vs No)	9.5 (5, 507087)	0.000	0.032
- Anxiety / stress related: Yes (vs No)	3 (5, 488139)	0.011	0.014
- Substance misuse: Yes (vs No)	3.8 (3, 70620)	0.009	0.011
- PDD / ID: Yes (vs No)	45.8 (1, 1206033)	0.000	0.029

Supplementary Table 2 (supplement to main document Table 3). Supplementary results from bias-reduced Logistic Regression analysis on **suicide attempt-related hospitalisation in people with epilepsy.** Model fit statistics by target risk factor including the D3 likelihood ratio test for multiply imputed data and average R2 value across imputed data sets.

Term	D3	p	R2
Age at SA	2.8 (1, 248796)	0.094	0.002
Age at SA (Non-linear)	9.4 (2, 318133)	0.000	0.012
Gender: Female (vs Male)	4.7 (1, 255993)	0.030	0.004
Ethnicity: White, Asian, Black, Mixed, Other (vs White)	7.4 (1, 1010)	0.007	0.006
Any neuropsychiatric comorbidities: Yes (vs No)	15.6 (3, 45193)	0.000	0.038
- Depression / Bipolar: Yes (vs No)	9.3 (5, 721896)	0.000	0.033
- Anxiety / stress related: Yes (vs No)	3.1 (5, 513571)	0.009	0.011
- Substance misuse: Yes (vs No)	3.5 (3, 44267)	0.014	0.008
- PDD / ID: Yes (vs No)	33.8 (1, 202937)	0.000	0.020
Previous SI: Yes (vs No)	11.9 (4, 161049)	0.000	0.038

Supplementary Table 3 (supplement to main document Table 4). Supplementary results from bias-reduced Logistic Regression analysis on **suicidal ideation in people with FDS.** Model fit statistics by target risk factor including the D3 likelihood ratio test for multiply imputed data and average R2 value across imputed data sets.

Term	D3	p	R2
Age at SI	56.3 (1, 3424225)	0.000	0.054

**Term**	**D3**	**p**	**R2**
Age at SI (Non-linear)	28.6 (3, 6658039)	0.000	0.088
Gender: Female (vs Male)	1.7 (1, 2810711)	0.191	0.002
Ethnicity: White, Asian, Black, Mixed, Other (vs White)	18 (1, 14485)	0.000	0.020
Any neuropsychiatric comorbidities: Yes (vs No)	8.8 (3, 739768)	0.000	0.028
- Depression / Bipolar: Yes (vs No)	5.6 (5, 7080170)	0.000	0.030
- Anxiety / stress related: Yes (vs No)	5.7 (5, 6035580)	0.000	0.031
- Personality Disorder: Yes (vs No)	8.7 (3, 696693)	0.000	0.029

Supplementary Table 4 (supplement to main document Table 5). Supplementary results from bias-reduced Logistic Regression analysis on **suicide attempt-related hospitalisation in people with FDS.** Model fit statistics by target risk factor including the D3 likelihood ratio test for multiply imputed data and average R2 value across imputed data sets.

**Term**	**D3**	**p**	**R2**
Age at SA	112.1 (1, 1350724)	0.000	0.118
Age at SA (Non-linear)	45.2 (3, 1962553)	0.000	0.157
Gender: Female (vs Male)	0 (1, 1019202)	0.920	0.000
Ethnicity: White, Asian, Black, Mixed, Other (vs White)	6.3 (1, 130198)	0.012	0.005
Any neuropsychiatric comorbidities: Yes (vs No)	6.9 (3, 13861242)	0.000	0.017
- Depression / Bipolar: Yes (vs No)	3.4 (5, 2532484)	0.004	0.013
Previous SI: Yes (vs No)	5.9 (4, 3672085)	0.000	0.020
